# Impact of Clove Essential Oil on the Intestinal Flora in Mice Infected with *Candida albicans*

**DOI:** 10.3390/molecules30112372

**Published:** 2025-05-29

**Authors:** Yuan Gao, Yuyang Guo, Shurong Chen, Jianmei Zhang, Jinhe Wang, Fuling Wang, Jianghan Luo, Lijun Yan

**Affiliations:** School of Pharmacy, Engineering Research Center of Natural Antineoplastic Drugs, Ministry of Education, Harbin University of Commerce, Harbin 150076, China; 18846086109@163.com (Y.G.); chensr0316@163.com (S.C.); zjm20010619@163.com (J.Z.); wangjinhe2025@163.com (J.W.); iwfl86@163.com (F.W.); 102690@hrb.edu.cn (J.L.); ylj@hrbcu.edu.cn (L.Y.)

**Keywords:** clove essential oil, *Candida albicans*, biofilm, intestinal flora, inflammation

## Abstract

This article investigates the antifungal activity of clove essential oil (CEO) against *Candida albicans*, as well as its inhibitory effects on *C. albicans* biofilm formation and the associated developmental processes. Furthermore, it evaluates the therapeutic efficacy of CEO in a mouse model of intestinal *C. albicans* infection and explores its impact on intestinal microbiota. Additionally, 16S rRNA high-throughput sequencing was conducted to analyze the alterations in the intestinal microbiota. The findings indicate that the administration of CEO in mice infected with *C. albicans* resulted in a normalization of body weight and an improvement in their symptoms. Histological analysis utilizing HE and PAS staining demonstrated that CEO exerted beneficial effects on the intestinal mucosal status of these infected mice. Furthermore, ELISA results revealed a dose-dependent reduction in the levels of IL-6, IL-8, and IL-17A within the small intestinal tissues of *C. albicans*-infected mice. Additionally, 16S rRNA gene analysis indicated that CEO effectively enhanced the richness and diversity within the intestinal microbiota of CEO treatment groups of mice that were investigated. Overall, CEO exhibits therapeutic potential against inflammation induced by intestinal *C. albicans* infection in mice. This effect can be attributed to its anti-inflammatory properties as well as its capacity to regulate the composition of intestinal flora.

## 1. Introduction

*Candida albicans* is a eukaryotic organism that is capable of existing in various forms, including yeast, fungal filaments, and pseudohyphae [1]. *C. albicans* is classified as a conditionally pathogenic fungus that can also inhabit healthy organisms. However, under conditions such as imbalances in the intestinal flora, compromised immunity, malnutrition, and other related factors, *C. albicans* has the potential to proliferate extensively. This proliferation may lead to increased invasion and virulence [2]. Consequently, it can result in mild superficial infections; however, severe cases may escalate to life-threatening deep infections. In China, the pathogens that are commonly responsible for intestinal fungal infections include Candida, Aspergillus, and Cryptococcus, among others, with *C. albicans* being the most prevalent [3,4]. Intestinal fungal infections belong to the category of “diarrhea” in Chinese medicine. At present, antifungal drugs such as fluconazole [5], clotrimazole [6], amphotericin B [7], and itraconazole [8,9], etc. are mostly used in clinical therapy, supplemented by probiotics. However, due to the obvious adverse reactions of many to specific antifungal drugs [10,11,12], as well as the serious gastrointestinal reactions caused by fungal mycelia and their high mortality rate, the treatment of intestinal fungal infections needs to be solved urgently.

Clove essential oil (CEO), derived from the dried flower buds of clove, exhibits a range of beneficial properties, including anti-inflammatory, antioxidant, and analgesic effects [13]. CEO is recognized as the most extensively studied secondary metabolite of clove. It has been certified as a safe dietary supplement by the Food and Drug Administration (FDA) and is widely utilized across various sectors, including the food industry, traditional medicine, and numerous industrial applications [14]. The active constituents of CEO encompass a diverse array of phenolic compounds, including eugenol, flavonoids, and terpenoids. The main component of CEO is eugenol (C_10_H_12_O_2_), which has obvious antimicrobial activities. Therefore, it is widely used in many fields including food production, pharmaceuticals, cosmetics, flavoring, and fragrances [15,16,17]. Notably, low concentrations (0.002–0.016% *v*/*v*) of CEO were shown to have no adverse effect on normal human dermal fibroblasts (NHDF) [18]. Further, the same study showed that eugenol selectively induced apoptosis in colon cancer cells (HCT-116, SW480, SW620) by activating caspase-3 and downregulating Bcl-2 without exhibiting significant toxicity to normal colon epithelial cells [19], highlighting its potential in the treatment of intestinal infections.

CEO had a certain inhibitory effect on the metabolic activity, colony morphology, and biofilm formation of *C. albicans* [20,21,22]. In addition, CEO also plays a certain role in improving immunity and regulating the intestinal flora. Some studies [23] have demonstrated the immunological regulation of CEO using Caco-2 cell line evaluation, and CEO was also shown to reduce the expression of Caco-2 cell line-related inflammatory genes and to reduce cytokine secretion in the absence of LPS. Also, studies have indicated that CEO can enhance the intestinal health of post-weaning piglets and promote their growth by reducing their hemolytic *E. coli* levels and improving their intestinal microenvironment [24]. The innovation of this investigation lies in the study of CEO on the intestinal tract when it is affected by *C. albicans* infection and intestinal flora disorder.

In this study, the anti-*C. albicans* effect of CEO was measured in vitro, and a mouse model of intestinal *C. albicans* infection in immunocompromised mice was established to evaluate whether intervention with different doses of CEO exerted therapeutic effects and intestinal microecological regulation. This work may provide a scientific basis for the clinical treatment of *C. albicans* infection in vitro and in vivo.

## 2. Results

### 2.1. Four Components Were Identified by GC-MS in CEO

Since a finished clove oil (eugenol) with a purity of 98% or more was used in this study, four components were identified in the GC-MS analysis report (Table 1). Eugenol was the main component, accounting for 81.52% of the peak area, and the other components were caryophyllene (14.45%), humulene (3.43%), and δ-cadinene (0.6%). After GC-MS analysis, the clear components of the CEO could be used for subsequent research and analysis. In addition, the deviations between the calculated RI and the reference of the NIST23 database are within ±15, and the mass spectral matching degree is >800, indicating that the compound identification results are reliable [25].

### 2.2. CEO Has an Inhibitory Effect on C. albicans

**Figure 1:**
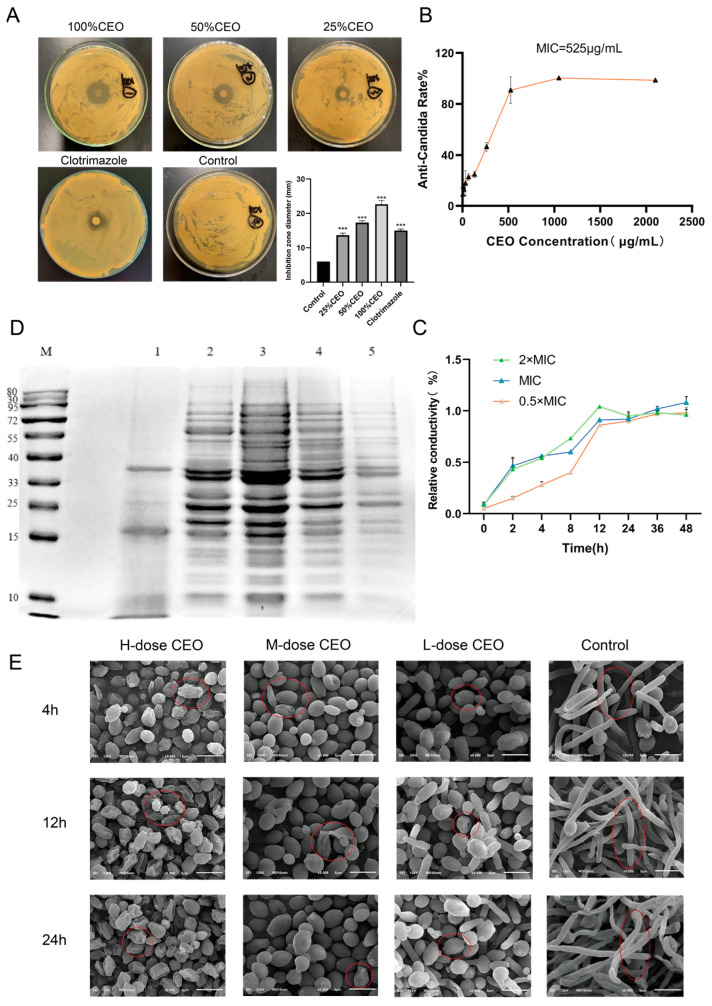
Diameter of anti-*C. albicans* zone of different concentrations of CEO (**A**), anti-Candida rate % for different concentrations of CEO after 48 h (**B**), effect of CEO on relative conductivity (**C**), effect of CEO on protein synthesis on *C. albicans* (**D**). The morphology of *C. albicans* at 4 h, 12 h, and 24 h was observed by scanning electron microscopy (**E**). Note: M: protein marker; 1: CEO 1050 μg/mL; 2: CEO 525 μg/mL; 3: CEO 262.50 μg/mL; 4: negative control; 5: clotrimazole. H-dose CEO: 1050 μg/mL, M-dose CEO:525 μg/mL, L-dose CEO: 262.5 μg/mL. Magnification: 5000× *** *p* < 0.001 compared to control group. The red circles in the CEO treatment group marked different yeast phase cells, while the control group labeled different hyphal cells.

In order to determine the inhibitory ability of CEO against *C. albicans*, inhibition zone and minimum inhibitory concentration (MIC) tests were carried out. The results of the inhibition zone experiment for CEO against *C. albicans* are shown in Figure 1A. Compared with the control group without the presence of an inhibition zone, the different concentrations of CEO had obvious antimicrobial zones, and the inhibition zone diameter increased with increases in the concentration. The diameter of the inhibition zone of the 25% CEO group (13.67 ± 0.58 mm) was relatively smaller than that of the clotrimazole group (15.04 ± 0.41 mm) (*p* < 0.001), while the diameters of the inhibition zone of the 50% CEO group (17.33 ± 0.58 mm) and 100% CEO group (22.67 ± 1.15 mm) were larger (*p* < 0.001), which showed that the CEO had a certain anti-*C. albicans* effect. As shown in Figure 1B, compared to the control group, the MIC of CEO against *C. albicans* was 525 μg/mL.

### 2.3. Effect of CEO on the Relative Conductivity of C. albicans

To evaluate the integrity of the biofilm of *C. albicans* in the CEO, a conductivity experiment was performed. The conductivity reflects the effect of the CEO on the membrane permeability of *C. albicans*. The conductivity experiment confirmed that, when CEO acts on the membrane of *C. albicans*, the membrane structure will be destroyed, resulting in the leakage of electrolytes such as Na^+^ and K^+^. Therefore, the relative variation level of the available conductivity value indirectly reflects the ability of CEO to cause damage to *C. albicans* and the strength of the damage.

The experimental outcomes illustrating the relative electrical conductivity of *C. albicans* subjected to CEO are presented in Figure 1C. Over the 48 h period, the concentration of 1050 μg/mL of CEO exhibited the most pronounced damage to the cell membrane, resulting in the rapid leakage of cell contents. In the initial 0–4 h, both the 1050 μg/mL and 525 μg/mL concentrations of CEO demonstrated the highest electrolyte leakage rate on *C. albicans*. Between 2 and 12 h, the electrolyte leakage from *C. albicans* displayed a dose-dependent relationship with the CEO concentration, with the relative conductivity increasing over the co-incubation period. Within 24–48 h, the relative conductivity of the 1050 μg/mL concentration group showed a slight downward trend. This concentration is the minimum bactericidal concentration which inhibits growth of *C. albicans*, reduces the number of bacteria, and decreases electrolyte leakage, thus leading to a decrease in relative conductivity; the relative conductivity of the 525 μg/mL and 262.50 μg/mL concentration groups showed a slow upward trend. In addition to the effect of the drug itself, the presence of a small amount of bacterial autolysis cannot be ruled out as an interference factor.

### 2.4. Effect of CEO on Protein Damage in C. albicans

The degree of damage to proteins in biofilms indirectly reflects the functional status, integrity, or damage of biofilms. The effect of CEO on the total protein damage of *C. albicans* is shown in Figure 1D. Compared to the negative control group, the protein strips of CEO were more absent at 1050 μg/mL and 525 μg/mL, and some proteins were enhanced at 262.50 μg/mL, with the deletion effect being most obvious at 1050 μg/mL. It was concluded that the total protein synthesis of *C. albicans* could be significantly affected at the concentrations of 1050 μg/mL and 525 μg/mL. According to the experimental data that were obtained, GSC1 (23 kDa), CDC28 (33 kDa), HOG1 (44 kDa), ERG11 (55 kDa), and TEC1 (62 kDa) may be inhibited by CEO, resulting in the loss of protein bands. As a result, CEO causes protein damage within the biofilm of *C. albicans*, resulting in membrane damage and abnormal membrane function.

### 2.5. Effects of CEO on Biofilm Formation

SEM was used to observe the biofilm formation stage of *C. albicans*, and the treatment effect of CEO was directly observed. The effects of CEO on the morphology of *C. albicans* at 4 h is shown in Figure 1E. In the negative control group, a few yeast phase cells can be seen, with a large number of mycelia and pseudo-mycelia also being formed. Compared to the negative control group, in the 262.50 μg/mL CEO group, there was no obvious hyphal formation; most of the cells remained round or round-like yeast phase, and a few began to extend into rod-like cells. In the 525 μg/mL CEO group, no hyphae were formed, and yeast cells were observed. In the 1050 μg/mL CEO group, there was no hyphal formation, and the yeast phase cells showed obvious shrinkage and depression.

The hyphal morphology at 12 h is shown in Figure 1E. A large number of hyphal cells and pseudohyphae were formed in the visual field of the control group, and were interwoven into a network. Compared with the control group, a few yeast cells were found to be dented in the 262.50 μg/mL CEO group, and pseudohyphae cell morphology began to appear at this concentration; the 525 μg/mL CEO group was observed to contain yeast phase cells, with obvious depression and wrinkles; the 1050 μg/mL CEO group had significant wrinkles compared to the adhesion period.

The state of *C. albicans* hyphae sheath formation at 24 h (Figure 1E) was as follows: Compared to the interwoven hyphae in the control group, the 262.50 μg/mL CEO group had a small number of short hyphae, with some yeast cells showing wrinkling and indentation on their surfaces. The 525 μg/mL CEO group and the 1050 μg/mL CEO group showed no hyphae formation, and the severe wrinkling and indentation in the 1050 μg/mL CEO group were dose-dependent.

### 2.6. Effect of CEO on General Signs and Body Weight of Mice

**Figure 2:**
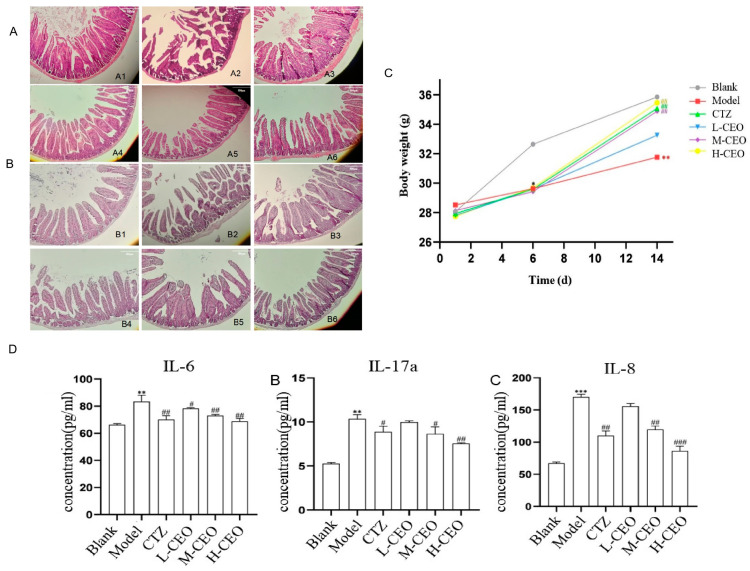
Effect of CEO on intestinal histopathology of *C. albicans*-infected mice. HE staining (**A**), PAS staining (**B**). Note: No. 1–6: blank group, model group, clotrimazole group, CEO low-, medium-, and high-dose group; effect of CEO on body mass changes in mice infected with *C. albicans* (**C**); effect of CEO on the content of IL-6, IL-17A, and IL-8 in intestinal tissues of *C. albicans*-infected mice (**D**). Note: results were expressed as mean ± SD. * *p* < 0.05, ** *p* < 0.01, *** *p* < 0.001; ^#^ *p* < 0.05, ^##^ *p* < 0.01, ^###^ *p* < 0.001 compared to blank group.

The effect of CEO on the general signs and body weight of intestinally infected *C. albicans* mice is a visual reflection of the treatment effect. The mice in the blank group were active, had a normal diet, and had shiny fur. Except for the mice in the blank group, the mice in the other groups showed decreased food intake, scraggly fur, withered fur, lethargy, soft and shapeless feces, and emaciation. After treatment with CEO, compared with the model group, the treatment group showed a certain improvement in their degree of sluggishness, their degree of reduced food intake, and their degree of soft and unformed feces. The effect of CEO on the change in body weight of the *C. albicans*-infected mice is shown in Figure 2C. On the first day of the experiment, there was no significant difference in the body weights of the mice in each group. Before infection with *C. albicans*, that is, on the sixth day of the experiment, compared to the blank group of mice, the body weights of the mice in each group showed a downward trend (*p* < 0.05). After treatment with CEO, that is, on the 7th–14th days of the experiment, compared to the model group of mice, the body weights of the mice in the high-dose CEO (H-CEO) treatment group and the medium-dose CEO (M-CEO) treatment group were significantly increased (*p* < 0.01). Compared with the blank group of mice, the body weights of the mice in the model group were significantly reduced (*p* < 0.01). The H-CEO group regained body weight and was better in this regard than the positive drug group.

The changes in the number of *C. albicans* in the intestinal lavage fluid of the infected mice during CEO treatment are shown in Table 2. At 5 days after the administration, the number of *C. albicans* in the lavage fluid of the treatment group showed a significant decrease and a dose-dependent decrease, suggesting that CEO had a therapeutic effect on the intestinal infection of *C. albicans*.

### 2.7. Effect of CEO on Intestinal Histopathology in Mice

HE and PAS pathological staining were performed to observe the histomorphological changes in the intestinal tract of *C. albicans*-infected mice. The HE staining results of the mouse intestinal tissues are shown in Figure 2A. In the blank group, the intestinal mucosa was intact, and the glands were arranged in an orderly way; in the model group, the intestinal mucosa was destroyed, the goblet cells were greatly reduced, most of the crypts were lost, the glandular structure was missing, inflammation infiltrated to the point that the submucosal muscularis was edematous, and the tissue damage was severe. After CEO treatment, the amount of goblet cells increased significantly, the crypt state recovered, inflammatory infiltration improved, and the CEO could reduce histopathological scores in a dose-dependent manner. Except for the clotrimazole-positive group, the H-CEO group had the best effect, indicating that CEO could improve the tissue damage caused by *C. albicans* infection.

The results of the PAS staining of the mouse intestinal tissues are shown in Figure 2B. More hyphae and spores were seen in the intestinal mucosa of the model group. Compared to the model group, the number of *C. albicans* spores and mycelia in the intestines of the CEO treatment group was reduced, and the treatment effect in the H-CEO group was more significant, with almost no *C. albicans* being present.

### 2.8. Effect of CEO on IL-6, IL-17A, and IL-8 in Mouse Intestinal Tissues

The level of inflammatory factors was detected to evaluate the ability of CEO to act on inflammation. The ELISA results for IL-6, IL-17A, and IL-8 are shown in Figure 2D. Compared to the blank group, the IL-6, IL-17A, and IL-8 contents increased significantly in the model group (*p* < 0.01), while those in the treatment group were lower than in the model group and determined to be most effective in the H-CEO group (*p* < 0.01); the difference was statistically significant for all groups.

### 2.9. High-Throughput Sequencing of the Mouse Gut Microbiota

16S rRNA high-throughput sequencing was used to analyze intestinal microbiota changes. The resulting sequences were optimized and their taxonomy was analyzed using the i-sanger cloud platform to count the community composition of each sample at different taxonomic levels and compare the similarity and difference relationships between them. At the same time, the degree and diversity (α-diversity) could be evaluated by single sample analysis.

### 2.10. α Diversity Analysis

The α diversity is the analysis of the species diversity in a single sample, and includes the Ace, Chao, Shannon, and Simpson indices. The Ace and Chao indices reflect the community richness of the species in a single sample, while the Shannon and Simpson indices reflect the microbial community diversity.

As shown in Table 3, the Chao, Ace, and Shannon indices in the treatment group were higher than those in the model group of *C. albicans* infection and were dose-dependent. The Chao and Ace indices in the H-CEO group and clotrimazole group were higher than those in the blank group. In conclusion, it is indicated that CEO can significantly (*p* < 0.01) increase the richness of intestinal microbiota. The change in the Shannon index indicates that CEO can improve the diversity of intestinal microbiota. Furthermore, the Simpson index was larger for the model group than the blank group, indicating that the diversity of the microbiota in the model group decreased, while the Simpson index of the CEO group and the clotrimazole group decreased significantly (*p* < 0.05), indicating that the diversity of the microbiota in the CEO group and the clotrimazole group increased.

### 2.11. Analysis of the OTU Species Situation

**Figure 3:**
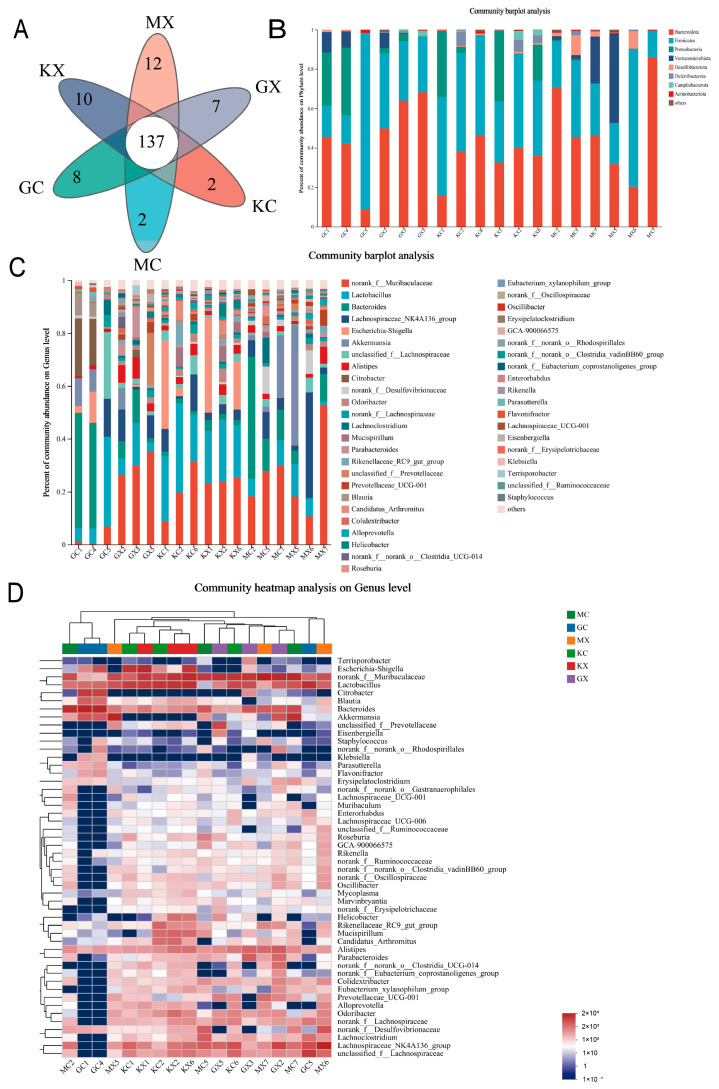
Venn diagram of the species composition of each group of intestinal flora (**A**); structural comparison at phylum level (**B**) and genus level, (**C**) and random forest analysis at species level (**D**). Note: GC, GX: H-CEO female, male group; MC, MX: M-CEO female, male group; KC, KX: blank female, male group.

In this study, a Venn diagram of OTUs was used to analyze the overlap in the number of OTUs and the types of OTUs between groups. The Venn diagram is shown in Figure 3A. Principal component analysis (PCA) was used to detect overall differences in bacterial abundance between samples. Overall, the H-CEO group had the highest number of OTUs, followed by the M-CEO group, and finally the blank group. The results indicated that the CEO increased the richness of the intestinal microbiota in a dose-dependent manner.

The results of the species analysis are shown in Figure 3B. At the phylum taxonomic level, eight phyla were detected at the door classification level; of these, Bacteroides and Firmicutes accounted for the largest proportion. Compared to that in the blank group, the abundances of Bacteroidota in the M-CEO group and the H-CEO group were generally increased, while the abundances of Firmicutes were decreased, so that ratio of Firmicutes/Bacteroidetes (F/B) in the CEO group was decreased and the microbiosis was hypobiosis, which was speculated to be due to inflammatory bowel disease caused by intestinal infection by *C. albicans*. Meanwhile, the abundance of Bacteroidota in the H-CEO group was lower than that in the M-CEO group, and the abundance of Firmicutes microbiota in H-CEO group was higher than that in the M-CEO group; thus, the F/B value of the H-CEO group was higher than that of the M-CEO group, indicating that CEO had the ability to improve intestinal inflammation.

The species analysis at the genus taxonomic level showed that the dominant genus in the control group was norank_f__Muribuculacease, and the abundance proportion of this group was the largest, followed by Lactobacillus. Compared to the blank group, the abundance of Lactobacillus and Escherichia-Shigella in the CEO treatment group was reduced, while the abundance of Bacteroides, Akkermansia, and Citrobacter bacteria was increased. Among the groups, there were more Lactobacillus and Escherichia-Shigella in the H-CEO group than in the M-CEO group, and the abundance of the H-CEO group approached that of the blank group, which showed that CEO could increase the abundance of beneficial bacteria. In addition, the abundance of norank_f__Muribuculacease microbiota in females in the CEO treatment group was significantly less than that in males. However, there were more Bacteroides and Lactobacillus in the females than in males, as shown in Figure 3C. The results of the multilayer clustering analysis are shown in Figure 3D.

**Figure 4:**
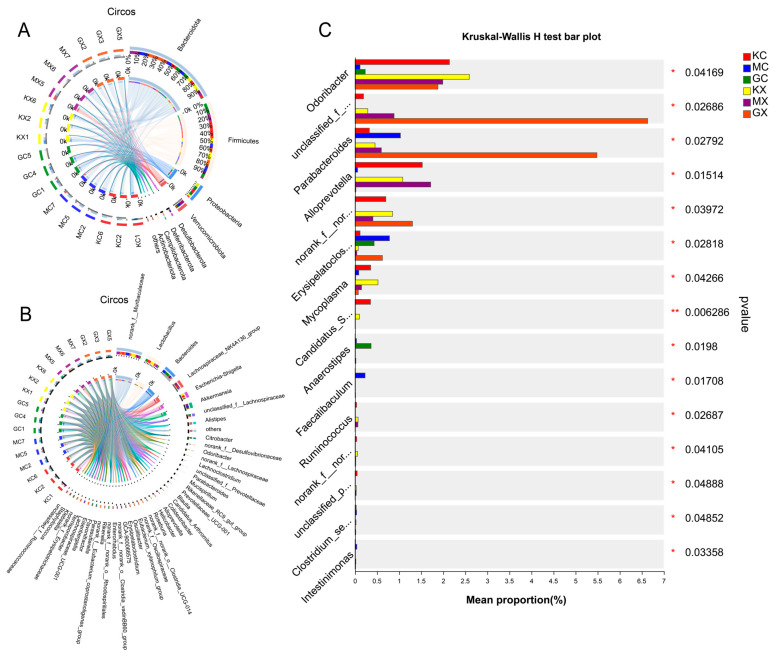
The intestinal flora was analyzed by Circos at phylum (Phylum) (**A**), and the right panel is Circos at genus (Genus) (**B**). Test for species variability in microbial communities between groups at the genus level (**C**). Note: GC, GX: H-CEO female, male group; MC, MX: M-CEO female, male group; KC, KX: Blank female, male group. * *p* < 0.05, ** *p* < 0.01, compared to control group.

Circos Graph is a data visualization tool. Figure 4 shows the different phylum and genus levels, as well as the abundance ratios of different groups in different microflora, by combining the species analyses at the genus and the phylum taxonomic levels. For example, in Figure 4A, the proportion of M-CEO and H-CEO groups in the Bacteroidota sample was larger than that of the blank group, and the proportion of the M-CEO group was larger. In the Firmicutes sample, the proportion of the M-CEO and H-CEO groups was lower than that of the blank group, and that of the medium dose group was lower. According to the proportion estimate, the order of the F/B values was blank > H-CEO > M-CEO group. This indicates that CEO can increase the F/B value and improve intestinal ecological imbalance.

### 2.12. β Diversity Analysis

**Figure 5:**
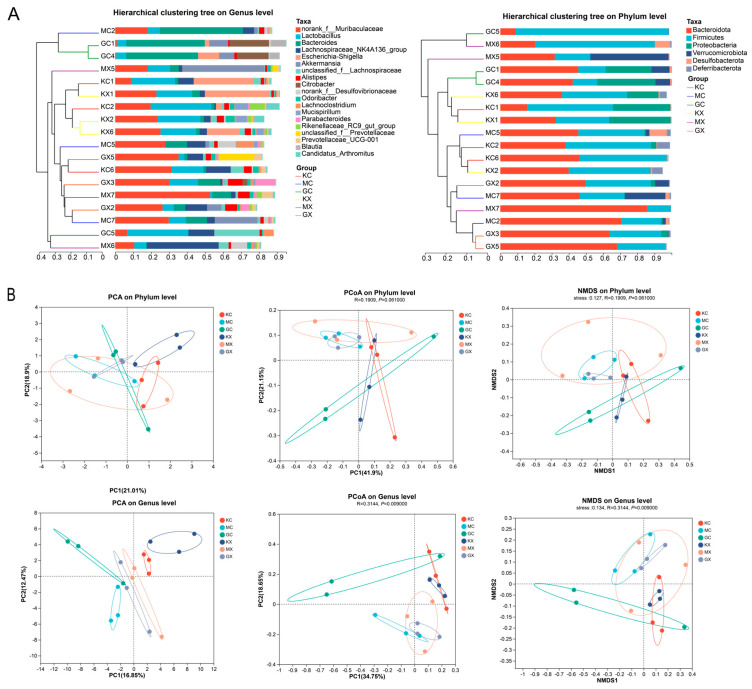
Cluster analysis of intestinal microflora in each sample at phylum and genus level (**A**). Coordinate analysis of each group in PCA; composition analysis of each group in PCoA; and non-metric multidimensional scale analysis (**B**). Note: GC, GX: H-CEO female, male group; MC, MX: M-CEO female, male group; KC, KX: blank female, male group.

The results of the cluster analysis of samples from different groups showed that the abundance distribution of species varies at the phylum and genus levels between the groups, and the abundance distribution of species also varies within each group, as shown in Figure 5A. The results of the sample cluster analysis, principal component analysis (PCA), and principal coordinate analysis (PCoA) are shown in Figure 5B. The differences between the groups were greater than those within the groups. The CEO and blank groups were clustered on different branches, and their sample points were distant from each other, indicating differences in community composition between the two groups. In the PCA, we observed that the sample points in the H-CEO group were closer to those in the blank group than those in the M-CEO group. Among the groups, the male sample points were more obvious in the CEO group. In conclusion, each drug treatment group can regulate the balance of an intestinal microbiota that was infected with *C. albicans*.

### 2.13. Association of Species Differences in the Mouse Intestinal Microbiota

The significance of the different species was evaluated based on the community abundance differences of the different grouped microbial samples. The inter-species variation of the top 15 species was determined based on the genus level, the results of which are shown in Figure 4C. The numbers of Odoribacter, Parabacteroides, unclassified_f__Prevotellaceae, and Erysipelatoclostridium bacterial groups in the H-CEO group were higher than those in the M-CEO group and less than those in the blank group. However, in the genus Alloprevotella, the H-CEO group was small. In addition, the abundances of Odoribacter, unclassified_f__Prevotellaceae, and norank_f__norank_o__Clostridia_UCG-014 were greater in the males than in the females in each group. Overall, the difference between the CEO and blank groups was relatively small, and the H-CEO group was better than the M-CEO group, indicating that CEO has an improvement effect on the intestinal flora of *C. albicans*-infected mice.

## 3. Discussion

The CEO component was analyzed by GC-MS. Eugenol, caryophyllene, humulene, and δ-cardinene, which are found in CEO, have shown significant potential against *C. albicans* infection through exerting multi-target synergistic effects, probably through antifungal [26] and anti-inflammatory properties [27], causing membrane disruption [28,29], and regulating intestinal microecology [30]. CEO’s anti-*C. albicans* activity was assessed, including the determination of the inhibition zone test and the MIC test. The experimental results showed that CEO could inhibit the growth of *C. albicans*, and that its MIC80 was 525 μg/mL. Next, conductivity experiments were carried out to determine that CEO could destroy the integrity of the *C. albicans* biomembrane, resulting in the leakage of K, ATP, etc. The results of the protein damage experiments showed that CEO could cause the loss of some proteins of *C. albicans*, such as GSC1 (23 kDa), CDC28 (33 kDa), and HOG1 (44 kDa), which indirectly reflected the destruction of the biofilm. By intuitively observing the mycelial morphology of *C. albicans* after CEO treatment, we found that CEO could inhibit the formation of *C. albicans* biofilm. In short, in vitro experiments showed that CEO had an inhibitory effect on *C. albicans* and affected the formation and state of its biofilm [31].

In terms of cytokine regulation, CEO can decrease the levels of IL-6, IL-8, and IL-17A during *C. albicans* infection, reducing inflammation. The inflammatory factors IL-6 and IL-8 were analyzed, as were pharmacodynamic indicators, to explore the effect of CEO on intestinal infection by *C. albicans*. In intestinal fungal infections [32], *C. albicans* compromises the integrity of the intestinal mucosa, exacerbating both fecal and tissue fungal loads and promoting the secretion of pro-inflammatory cytokines in the intestinal tissue. The occurrence of these *C. albicans* infections caused an increase in the IL-6 and IL-8 levels. Studies have shown that the intestinal flora can play a major immune protective role in the invasion of *C. albicans* through exerting Th17-type cell immunity. IL-17A is a characteristic cytokine secreted by Th17 cells and plays an important function in the anti-infection immunity of the host [33]. Studies have shown that a significant decrease in IL-17A secretion by Th17 cells was observed after the antifungal agent nystatin was used, which played a role in the treatment of alcoholic liver injury caused by *C. albicans* [34]. In this study, CEO decreased the cytokines IL-6, IL-8, and IL-17A, which were elevated by intestinal infection, and improved inflammatory status.

CEO can also improve intestinal microbiosis and promote the recovery of intestinal inflammation. The observed change in the α diversity analysis index indicates that CEO can increase the richness and diversity of intestinal microbiota, thus improving the intestinal microbiota imbalance caused by intestinal infection. The results of the species analysis showed that CEO increased the reduction in the F/B ratio caused by intestinal infection with *C. albicans*, and that CEO had the ability to improve intestinal inflammation. Dysregulation of the F/B ratio links gut dysbiosis to disease. A high F/B ratio is usually associated with diseases such as obesity [35], hypertension [36], and prostatic hypertrophy [37], while a low F/B ratio is usually associated with inflammatory bowel disease. The findings of the species analysis show that the abundance of Lactobacillus in the H-CEO group was lower than that in the blank group, while the abundance of Akkermansia increased. Bidirectional crosstalk mediated by Lactobacillus may promote anti-inflammatory responses and thus improve inflammation occurring in the gastrointestinal tract [38]. As a beneficial bacterium, Akkermansia will gradually decrease with the development of enteritis. Akkermansia can regulate the immune response of the spleen and intestine, and the higher abundance of microflora observed in the treatment group was shown to better reduce the level of inflammation in the intestine [39]. Therefore, the CEO group has the effect of improving intestinal inflammation. The analysis of the differences in the microbial communities observed in the different groups, shown in Figure 4C, showed that the abundance of Odoribacter and unclassified-f-Prevotellaceae was higher in the male group, while the abundance of Bacteroides and Lactobacillus was lower, indicating gender differences in intestinal microbiota. In addition, the intestinal flora is also affected by age, location, and other factors. Therefore, it is essential to take into account multiple factors in detail when conducting experimental studies and clinical interventions to obtain a more complete picture of microbiome dynamics [40]. In summary, CEO significantly and effectively improved the flora imbalance, and adjusted the recovery of the flora to a state close to normal, in mice.

## 4. Materials and Methods

### 4.1. Strain, Animals, and Chemicals

*Candida albicans* strain SN250 was generously provided by Professor Chen Changbin. ICR mice, SPF grade, weighing 20 ± 2 g, with half being male and half being female, were purchased from Yisi Laboratory Animal Technology Co., Ltd. (Changchun, China); and tested by the Jilin Provincial Laboratory Animal Quality Inspection Center, with the license number SCXK-2022-0001. This experiment has been approved by the Laboratory Animal Ethics Committee of Harbin University of Commerce, with the number HSDYXY-2022014. Clotrimazole was purchased from Jinsui Biotechnology Co., Ltd. (Shanghai, China); streptomycin sulfate was purchased from Lukang Pharmaceutical Co., Ltd. (Jining, China); cyclophosphamide was purchased from Hengrui Pharmaceutical Co., Ltd. (Lianyungang, China); clove oil was purchased from Vicky Biotechnology Co., Ltd. (Chengdu, China).

### 4.2. GC-MS Analysis

The instrument used for analysis of CEO was gas chromatography-mass spectrometry (GC-MS) (7890A-5975C, Agilent Technologies, Santa Clara, CA, USA). Analysis was performed using a GC-MS system equipped with an HP-5MS column (30 m × 0.25 mm, 0.25 μm, Agilent Technologies, USA). The initial column temperature was set at 50 °C and then gradually ramped up to 200 °C at a rate of 5 °C/min. Subsequently, the temperature was raised to 280 °C at a rate of 10 °C/min and maintained for 10 min. The carrier gas was helium (He) with a flow rate of 1.0 mL/min. Injection temperature was 280 °C. The ionization mode was EI+, the electron energy was 70 eV, the ion source temperature was 250 °C, and the quadrupole temperature was 150 °C. In addition, the retention index (RI) of the components was calculated to match the reference RI value in NIST23 EI-MS database to identify the components [41].

### 4.3. Anti-C. albicans Assay

According to the method specified in the M-44A protocol of the National Clinical and Laboratory Standards Institute (CLSI) of the United States, the inhibitory zone diameter of the CEO against *C. albicans* was determined. A sterile filter paper (d = 6 mm) was fully soaked in the CEO, clotrimazole (5 mg/mL), and PBS for 5 min, and divided into treatment group, positive drug control group, and negative group. The CEO ratio was diluted to 50% CEO and 25% CEO with normal saline. A 100 μL fungal suspension was inoculated on the YPD medium. Filter paper pieces treated in different groups were dried at room temperature and attached to the center of the petri dish. After incubation at 30 °C for 24 h, the diameter of the antimicrobial zone was measured with a vernier caliper.

Following the micro-dilution method in the M27-A protocol established by the National Committee for Clinical Laboratory Standards (NCCLS), the concentration of the fungal solution was adjusted to 2 × 10^3^ CFU/mL. The CEO and clotrimazole were dissolved in a small amount of DMSO to serve as the stock solution. For in vitro assays, the stock was further diluted with yeast peptone dextrose (YPD) liquid medium to achieve the desired concentrations. CEO was diluted into 10 concentrations: 2100, 1050, 525, 262.50, 131.25, 65.63, 32.81, 16.41, 8.20, and 4.10 μg/mL with normal saline, and clotrimazole was diluted to 10 concentrations: 5, 2.50, 1.25, 0.63, 0.31, 0.10, 0.078, 0.039, 0.020, and 0.0098 μg/mL. A total of 100 μL of different concentrations of CEO and clotrimazole were added to 96-well plates sequentially, while the negative control group contained an equivalent volume of saline [42]. YPD solution without fungal suspension was added to the blank wells, and 100 μL fungal suspension was added to the other wells. Then, the plates were incubated at 30 °C for 48 h, and the OD_630_ nm was measured to assess fungal MIC_80_, which represents the minimum concentration of CEO that inhibits *C. albicans* growth by 80%.

### 4.4. Conductivity Experiment

The fungal suspension (2 × 10^6^ CFU/mL) was combined with various concentrations of CEO (0.5 × MIC, MIC, 2 × MIC), while a group without CEO served as the blank control. After thorough mixing, the resulting value was recorded as A and the mixture was incubated at 30 °C. Measurements of electrical conductivity (denoted as B) were taken at 0, 2, 4, 8, 12, 24, 36, and 48 h of co-incubation. Following each time point, the blank control group tube was subjected to boiling water for 10 min, and its electrical conductivity (denoted as C) was measured after it reached a constant temperature. The solvent conductivity value (denoted as D) was also recorded. These procedures were repeated three times [43,44].

The relative conductivity was calculated as follows:

relative conductivity (%) = (B − A) ÷ (C − D) × %(1)

### 4.5. Protein Damage Test

Various concentrations of CEO (0.5 × MIC, MIC, 2 × MIC) were introduced into *C. albicans* suspensions during the later stage of logarithmic growth. Subsequently, total protein extraction was performed using the Total Protein Extraction Kit (EX2531, Solarbio, Beijing, China). The process involved the addition of wall-breaking solution I, suspension of the fungus on ice for 5 min, and centrifugation at 12,000 rpm at 4 °C for 2 min. Following the discarding of the supernatant, wall-breaking solution II (500 μL) was added and the mixture was incubated on ice for an additional 5 min. After a 2 min centrifugation at 12,000 rpm at 4 °C, the supernatant was discarded, and 500 μL of lysate was added and left at room temperature for 60 min. Following a 10 min centrifugation at 12,000 rpm at 4 °C, protein concentration was determined using the Lowry method. Adjusting it to the same concentration, the protein sample was treated with buffer (5×), heated at 100 °C for 5 min for denaturation, and subsequently removed. SDS-PAGE was conducted using a 5% concentrated gel and 12% separation gel. The gel was stained with Coomassie Brilliant blue, and the total protein changes were observed and recorded.

### 4.6. Observation of C. albicans Biofilm Formation Stage by SEM

*C. albicans* suspension (2 × 10^6^ CFU/mL) was mixed with CEO concentration at 2 × SMIC_80_, SMIC_80_, and 0.5 × SMIC_80_, and incubated at 37 °C for 4, 12, and 24 h. The suspensions were centrifuged at 3000 rpm for 3 min, and the precipitate was dissolved in 600 μL of a 2.5% glutaraldehyde solution and stored at 4 °C overnight, during which time it was protected from light. After discarding the glutaraldehyde solution, *C. albicans* were washed with PBS 3 times, followed by gradient dehydration with 30%, 50%, 70%, 90%, and 100% ethanol for 15 min. The sample was then dried in a vacuum dryer overnight. The *C. albicans* samples were then vacuumed, gilded, and observed under 5000-times magnification using scanning electron microscopy (SEM) (JEOL, Tokyo, Japan) [15,45,46].

### 4.7. Construction of a Mouse Intestinal Model of C. albicans Infection

Seventy-two ICR mice were randomly divided into 6 groups, including a blank group, a clotrimazole group, a model group, and low-, medium-, and high-dose treatment groups. Except for the blank group, mice in each group were given intragastric streptomycin sulfate [47] (100 mg/kg, 1 mL/mouse) and intraperitoneal cyclophosphamide [48,49] (50 mg/kg, 1 mL/mouse) once a day for 5 consecutive days, while mice in the blank group were treated with saline at the same frequency. After 12 h of intervention on the 5th day, mice in each group except the blank group were given intragastric *C. albicans* suspension (1.6 × 10^9^ CFU/L, 0.5 mL/mouse) for 1 day, in order to establish the ICR mouse model of *C. albicans* intestinal infection. The successful establishment of a mouse intestinal model of *C. albicans* infection was associated with changes in the intestinal microbiota [50]. The signs of the mice in each group were observed daily, and the weight of the mice was recorded at a fixed time. On the 7th day, clotrimazole (20 mg/kg) was administered by gavage (0.2 mL/mouse) in the clotrimazole group, and different doses of CEO (300 mg/kg, 150 mg/kg, 75 mg/kg) were administered by gavage in the treatment group, while the blank group and the model group were treated with saline. The CEO was administered for 7 consecutive days.

Fresh fecal samples were collected within 24 h before euthanasia. After the mice were euthanized, the small intestines of the mice were collected, cleaned with PBS, and stored for the following experiment.

### 4.8. IL-6, IL-8, IL-17A Determination

Interleukin-6 (IL-6), interleukin-8 (IL-8), and interleukin-17A (IL-17A) were detected by double-antibody sandwich assay. The microplates were coated with purified antibodies, followed by the addition of IL-6, IL-8, and IL-17A standards, and then the corresponding antibodies, which were labeled by peroxidase (HRP), were used. The substrate TMB was added to the antibodies to develop color after incubation and washing. TMB was catalyzed by HRP enzymes to appear blue, and was converted to the final yellow color by interaction with the acid color development stop solution. The color in the microplate was correlated with either IL-6, IL-8, or IL-17A. The absorbance value at 450 nm was measured using a microplate reader, and the concentrations of IL-6, IL-8, and IL-17A in the supernatant of the intestine tissue were determined based on a standard curve.

### 4.9. HE and PAS Staining

The small intestine tissues were placed in the embedding box, soaked in 10% formalin solution, and fixed at 4 °C for one week; the formalin solution was used for the subsequent staining experiments. The fixed intestine tissue was dehydrated by ethanol gradient, paraffin embedded, sectioned, stained by HE and PAS, and sealed by gum. Then, the ratio was adjusted under the microscope until appropriate and photographed.

### 4.10. Sequencing of Intestinal Flora of MICE Colonized by C. albicans

Microbial community genomic DNA was extracted from fecal samples and subsequently amplified via PCR using the primers 338F (5′-ACTCCTACGGGAGGCAGCAG-3′) and 806R (5′-GGACTACHVGGGTWTCTAAT-3′). The amplification aimed to detect the 16S rRNA gene within the V3–V4 hypervariable region. Sequencing was conducted on the Illumina MiSeq platform (Illumina, San Diego, CA, USA) in accordance with standard procedures established by Majorbio Bio-pharm Technology Co., Ltd. (Shanghai, China).

### 4.11. Data Statistics

The figures were mainly generated using GraphPad Prism 9.5; one-way ANOVA and two-way ANOVA were applied. *p* < 0.05 was statistically significant.

## 5. Conclusions

In conclusion, this study indicated the antifungal effect of CEO when it was combined with the treatment of *C. albicans* intestinal infection. It is found that CEO can not only improve intestinal fungal infection and reduce the inflammatory response, but that it can also effectively regulate the intestinal flora. The previous research on the effect of CEO on the intestinal flora was limited. Therefore, it is speculated that CEO may have potential applications in the treatment of intestinal infection, even enteritis and colitis [51], which also provides value for the study of clinical fungal infections in the future.

## Figures and Tables

**Table 1:** GC-MS analysis of clove oil.

Compound	CAS	Retention Time/min	Calculated RI	NIST23 RI Value	Peak Area %
Eugenol	97-53-0	20.812	1349	1357 ± 3	81.52
Caryophyllene	87-44-5	22.309	1422	1419 ± 3	14.45
α-Humulene	6753-98-6	23.181	1459	1454 ± 3	3.43
δ-cadinene	483-76-1	25.300	1519	1524 ± 2	0.60

**Table 2:** Effect of CEO on the amount of *C. albicans* in mouse intestinal lavage fluid ($\bar{X}$ ± s, n = 12).

Time	Group	Concentration mg/(kg·d)	CFU/10^4^
5 d	High-dose group	300	1.53 ± 0.26
	Medium-dose group	200	1.69 ± 0.37
	Low-dose group	100	1.83 ± 0.33
	Clotrimazole group	20	1.48 ± 0.21
	Model group	—	3.13 ± 0.19
	Control group	—	0 ± 0

**Table 3:** The Alpha diversity index table.

Index	Group						SE	*p*-Value
	Blank Group	Model Group	L-CEO Group	M-CEO Group	H-CEO Group	CZT Group		
Chao	386.62 ± 9.99 ^Aa^	135.61 ± 22.31 ^Bb^	371.24 ± 16.80 ^Cc^	380.16 ± 20.41 ^Cc^	420.14 ± 0.72 ^Cd^	428.51 ± 0.72 ^Cd^	27.56	<0.01
ACE	384.44 ± 8.18 ^Aa^	127.93 ± 11.87 ^Bb^	369.45 ± 7.45 ^Cc^	377.68 ± 22.90 ^Cc^	419.99 ± 12.20 ^Cd^	422.51 ± 12.20 ^Cd^	27.55	<0.01
Simpso	0.16 ± 0.01 ^Aa^	0.44 ± 0.03 ^Bb^	0.06 ± 0.01 ^Cc^	0.13 ± 0.02 ^Cd^	0.09 ± 0.01 ^Ce^	0.08 ± 0.01 ^Ce^	0.44	<0.01
Shanno	2.88 ± 0.55 ^Aa^	1.49 ± 0.37 ^Ab^	3.22 ± 0.56 ^Ac^	3.12 ± 0.74 ^Ac^	3.35 ± 0.52 ^Ac^	3.30 ± 0.52 ^Ac^	0.22	<0.05

Note: Groups that are significantly different from each other at *p* < 0.05 are indicated by different letters (A–C, a–e).

## Data Availability

The data used in the current study are available from the corresponding author upon reasonable request.

## References

[1] Lee J.H., Kim Y.G., Lee J. (2024). Antibiofilm activity of lawsone against polymicrobial enterohemorrhagic *Escherichia coli* O157:H7 and *Candida albicans* by suppression of curli production and hyphal growth. Phytomedicine.

[2] Lohse M.B., Gulati M., Johnson A.D., Nobile C.J. (2017). Development and regulation of single- and multi-species *Candida albicans* biofilms. Nat. Rev. Microbiol..

[3] Antonia L., Michelle M., Alexander S.M., Mark S.G., Bernhard H. (2021). In vitro infection models to study fungal–host interactions. FEMS Microbiol. Rev..

[4] Talapko J., Juzbai M., Matijevi T., Pustijanac E., Krlec I. (2021). *Candida albicans*—The Virulence Factors and Clinical Manifestations of Infection. J. Fungi..

[5] Daneshnia F., de Almeida Júnior J.N., Ilkit M., Lombardi L., Perry A.M., Gao M., Nobile C., Egger M., Perlin D., Zhai B. (2023). Worldwide emergence of fluconazole-resistant *Candida parapsilosis*: Current framework and future research roadmap. Lancet Microbe.

[6] Lertsuphotvanit N., Tuntarawongsa S., Jitrangsri K.P.T. (2023). Clotrimazole-Loaded Borneol-Based In Situ Forming Gel as Oral Sprays for Oropharyngeal Candidiasis Therapy. Gels.

[7] Chowdhary A., Jain K., Chauhan N. (2023). Candida auris Genetics and Emergence. Annu. Rev. Microbiol..

[8] Bassi R.C., Boriollo M.F.G. (2022). Amphotericin B, fluconazole, and nystatin as development inhibitors of *Candida albicans* biofilms on a dental prosthesis reline material: Analytical models invitro. J. Prosthet. Dent..

[9] Pfaller M.A., Carvalhaes C.G., Castanheira M. (2023). Susceptibility patterns of amphotericin B, itraconazole, posaconazole, voriconazole and caspofungin for isolates causing invasive mould infections from the SENTRY Antifungal Surveillance Program (2018–2021) and application of single-site epidemiological cutoff values to evaluate amphotericin B activity. Mycoses.

[10] Tragiannidis A., Gkampeta A., Vousvouki M., Vasileiou E., Groll A.H. (2021). Antifungal agents and the kidney: Pharmacokinetics, clinical nephrotoxicity, and interactions. Expert Opin. Drug Saf..

[11] Rakhshan A., Rahmati Kamel B., Saffaei A., Tavakoli-Ardakani M. (2023). Hepatotoxicity Induced by Azole Antifungal Agents: A Review Study. Iran. J. Pharm. Res..

[12] Draskau M.K., Rosenmai A.K., Scholze M., Pedersen M., Boberg J., Christiansen S., Svingen T. (2021). Human-relevant concentrations of the antifungal drug clotrimazole disrupts maternal and fetal steroid hormone profiles in rats. Toxicol. Appl. Pharm..

[13] Ben Hassine D., Kammoun El Euch S., Rahmani R., Ghazouani N., Kane R., Abderrabba M., Bouajila J. (2021). Clove Buds Essential Oil: The Impact of Grinding on the Chemical Composition and Its Biological Activities Involved in Consumer’s Health Security. BioMed Res. Int..

[14] Noazira Wan Adnan W., Ulfah Karim N., Husna Yusoff N.A., Ihwan Zakariah M., Hassan M. (2021). Effect of *Cymbopogon citratus* Essential Oil (EO) on Handling Stress in Giant Freshwater Prawn (*Macrobrachium rosenbergii*). Pak. J. Biol. Sci..

[15] Pandey V.K., Srivastava S., Ashish, Dash K.K., Singh R., Dar A.H., Singh T., Farooqui A., Shaikh A.M., Kovacs B. (2024). Bioactive properties of clove (*Syzygium aromaticum*) essential oil nanoemulsion: A comprehensive review. Heliyon.

[16] Shahina Z., Molaeitabari A., Sultana T., Dahms T.E.S. (2022). Cinnamon Leaf and Clove Essential Oils Are Potent Inhibitors of *Candida albicans* Virulence Traits. Microorganisms.

[17] Kiki M.J. (2023). In Vitro Antiviral Potential, Antioxidant, and Chemical Composition of Clove (*Syzygium aromaticum*) Essential Oi. Molecules.

[18] Prashar A., Locke I.C., Evans C.S. (2006). Cytotoxicity of clove (*Syzygium aromaticum*) oil and its major components to human skin cells. Cell Prolif..

[19] Wijewantha N., Sane S., Eikanger M., Antony R.M., Potts R.A., Lang L., Rezvani K., Sereda G. (2023). Enhancing anti-tumorigenic efficacy of eugenol in human colon cancer cells using enzyme-responsive nanoparticles. Cancers.

[20] Shahina Z., Ndlovu E., Persaud O., Sultana T., Dahms T.E.S. (2022). *Candida albicans* Reactive Oxygen Species (ROS)-Dependent Lethality and ROS-Independent Hyphal and Biofilm Inhibition by Eugenol and Citral. Microbiol. Spectr..

[21] El-Baz A.M., Mosbah R.A., Goda R.M., Mansour B., Sultana T., Dahms T.E.S., El-Ganiny A.M. (2021). Back to Nature: Combating *Candida albicans* Biofilm, Phospholipase and Hemolysin Using Plant Essential Oils. Antibiotics.

[22] Ahmad I., Farheen M., Kukreti A., Afzal O., Akhter M.H., Chitme H., Visht S., Altamimi A.S.A., Alossaimi M.A., Alsulami E.R. (2023). Natural Oils Enhance the Topical Delivery of Ketoconazole by Nanoemulgel for Fungal Infections. ACS Omega.

[23] Valdivieso-Ugarte M., Plaza-Díaz J., Gómez-Llorente C., Lucas Gómez E., Sabés-Alsina M., Gil Á. (2021). In vitro examination of antibacterial and immunomodulatory activities of cinnamon, white thyme, and clove essential oils. J. Funct. Foods.

[24] Luise D., Correa F., Negrini C., Virdis S., Mazzoni M., Dalcanale S., Trevisi P. (2023). Blend of natural and natural identical essential oil compounds as a strategy to improve the gut health of weaning pigs. Anim. Int. J. Anim. Biosci..

[25] McGlynn D.F., Yee L.D., Garraffo H.M., Geer L.Y., Mak T.D., Mirokhin Y.A., Tchekhovskoi D.V., Jen C.N., Goldstein A.H., Kearsley A.J. (2025). New Library-Based Methods for Nontargeted Compound Identification by GC-EI-MS. J. Am. Soc. Mass. Spectr..

[26] Biernasiuk A., Baj T., Malm A. (2022). Clove essential oil and its main constituent, eugenol, as potential natural antifungals against *Candida* spp. alone or in combination with other antimycotics due to synergistic interactions. Molecules.

[27] Viveiros M.M.H., Silva M.G., Da Costa J.G.M., de Oliveira A.G., Rubio C., Padovani C.R., Rainho C.A., Schellini S.A. (2022). Anti-inflammatory effects of α-humulene and β-caryophyllene on pterygium fibroblasts. Int. J. Ophthalmol..

[28] Didehdar M., Chegini Z., Shariati A. (2022). Eugenol: A novel therapeutic agent for the inhibition of *Candida* species infection. Front. Pharmacol..

[29] Qin R., Yang S., Fu B., Chen Y., Zhou M., Qi Y., Xu N., Wu Q., Hua Q., Wu Y. (2024). Antibacterial activity and mechanism of the sesquiterpene δ-cadinene against *Listeria monocytogenes*. LWT.

[30] Li M., Zhao Y., Wang Y., Geng R., Fang J., Kang S.G., Huang K., Tong T. (2022). Eugenol, a major component of clove oil, attenuates adiposity, and modulates gut microbiota in high-fat diet-fed mice. Mol. Nutr. Food Res..

[31] Sangeeta J.P., Aishwarya O.B., Omkar D.B., Madhura N.B. (2024). Anti-biofilm effect of clove oil against *Candida albicans*: A systematic review. J. Oral Maxillofac. Pathol..

[32] Ma K., Chen M., Liu J., Ge Y., Wang T., Wu D., Yan G., Wang C., Shao J. (2021). Sodium houttuyfonate attenuates dextran sulfate sodium associated colitis precolonized with *Candida albicans* through inducing β-glucan exposure. J. Leukoc. Biol..

[33] Liu Y., Tang B., Wang F., Tang L., Lei Y., Luo Y., Huang S., Yang M., Wu L., Wang W. (2020). Parthenolide ameliorates colon inflammation through regulating Treg/Th17 balance in a gut microbiota-dependent manner. Theranostics.

[34] Zeng S., Rosati E., Saggau C., Messner B., Chu H., Duan Y., Hartmann P., Wang Y., Ma S., Huang W.J.M. (2023). *Candida albicans*-specific Th17 cell-mediated response contributes to alcohol-associated liver disease. Cell Host Microbe.

[35] Cheng Z., Zhang L., Yang L., Chu H. (2022). The critical role of gut microbiota in obesity. Front. Endocrinol..

[36] Yang T., Santisteban M.M., Rodriguez V., Li E., Ahmari N., Carvajal J.M., Zadeh M., Gong M., Qi Y., Zubcevic J. (2015). Gut dysbiosis is linked to hypertension. Hypertension.

[37] Takezawa K., Fujita K., Matsushita M., Motooka D., Hatano K., Banno E., Shimizu N., Takao T., Takada S., Okada K. (2021). The Firmicutes/Bacteroidetes ratio of the human gut microbiota is associated with prostate enlargement. Prostate.

[38] Rastogi S., Singh A. (2022). Gut microbiome and human health: Exploring how the probiotic genus *Lactobacillus* modulate immune responses. Front. Pharmacol..

[39] Cani P.D., Depommier C., Derrien M., Everard A., de Vos W.M. (2022). *Akkermansia muciniphila*: Paradigm for next-generation beneficial microorganisms. Nat. Rev. Gastroenterol. Hepatol..

[40] Ortiz-Alvarez De La Campa M., Curtis-Joseph N., Beekman C., Belenky P. (2024). Gut biogeography accentuates sex-related differences in the murine microbiome. Microorganisms.

[41] NIST/EPA/NIH (2023). NIST Standard Reference Database 1A: NIST/EPA/NIH Mass Spectral Library (NIST 23). https://www.nist.gov/srd/nist-standard-reference-database-1a.

[42] Ma H., Zhao X., Yang L., Su P., Fu P., Peng J., Yang N., Guo G. (2020). Antimicrobial Peptide AMP-17 Affects *Candida albicans* by Disrupting Its Cell Wall and Cell Membrane Integrity. Infect. Drug Resist..

[43] Pavlin M., Kandušer M., Reberšek M., Pucihar G., Hart F.X., Magjarevićcacute R., Miklavčič D. (2005). Effect of cell electroporation on the conductivity of a cell suspension. Biophys. J..

[44] Li N., Gao C., Peng X., Wang W., Luo M., Fu Y., Zu Y. (2014). Aspidin BB, a phloroglucinol derivative, exerts its antibacterial activity against *Staphylococcus aureus* by inducing the generation of reactive oxygen species. Res. Microbiol..

[45] Farkash Y., Feldman M., Ginsburg I., Steinberg D., Shalish M. (2018). Green Tea Polyphenols and Padma Hepaten Inhibit *Candida albicans* Biofilm Formation. Evid.-Based Complement. Altern. Med..

[46] Panariello B.H.D., Klein M.I., Mima E.G.D.O., Pavarina A.C. (2018). Fluconazole impacts the extracellular matrix of fluconazole-susceptible and-resistant *Candida albicans* and *Candida glabrata* biofilms. J. Oral Microbiol..

[47] Duffy S.C., Lupien A., Elhaji Y., Farag M., Marcus V., Behr M.A. (2023). Establishment of persistent enteric mycobacterial infection following streptomycin pre-treatment. Gut Pathog..

[48] Ying M., Yu Q., Zheng B., Wang H., Wang J., Chen S., Nie S., Xie M. (2020). Cultured *Cordyceps sinensis* polysaccharides modulate intestinal mucosal immunity and gut microbiota in cyclophosphamide-treated mice. Carbohydr. Polym..

[49] Kim H., Hong J.Y., Lee J., Yeo C., Jeon W., Lee Y.J., Ha I. (2024). Immune-boosting effect of Yookgong-dan against cyclophosphamide-induced immunosuppression in mice. Heliyon.

[50] Koh A.Y. (2013). Murine Models of Candida Gastrointestinal Colonization and Dissemination. Eukaryot. Cell.

[51] Petrocelli G., Farabegoli F., Valerii M.C., Giovannini C., Sardo A., Spisni E. (2021). Molecules present in plant essential oils for prevention and treatment of colorectal cancer (CRC). Molecules.

